# Branchioma: A Classic Example

**DOI:** 10.1007/s12105-025-01805-y

**Published:** 2025-06-04

**Authors:** Melad N. Dababneh, Kaitlyn Ooms

**Affiliations:** 1 Department of Pathology, Heersink School of Medicine, University of Alabama at Birmingham, NP3552, 619 19th St S, Birmingham, AL 35249 USA; 2 Robert J. Tomsich Pathology & Laboratory Medicine Institute, Cleveland Clinic, Cleveland, OH USA

**Keywords:** Branchioma, Suprasternoclavicular

## Abstract

Branchioma is a very rare benign neoplasm of the neck. This case highlights its classic clinical presentation, histomorphology and immunoprofile.

Branchioma, also referred to as biphenotypic branchioma, is a benign neoplasm with almost exclusive predilection to the lower anterior neck at the area of sternoclavicular junction. It is thought to arise from branchial pouch derivatives, and histologically displays a mixed proliferation of spindled, epithelial and adipose tissue [1, 2]. Prior terminology of this entity includes ectopic hamartomatous thymoma and thymic anlage tumor, but branchiomas have no true thymic differentiation [2]. 

This is a case presentation of a middle-aged male patient with a relatively firm suprasternoclavicular mass. Histologic sections of the resection specimen show a well circumscribed neoplasm, composed of predominant spindled cells, along with epithelial islands and scattered mature adipose tissue (Fig. 1). The plump spindled cells have variable cytology and architecture, including amphophilic, eosinophilic and clear cytoplasm, and are arranged in fascicular or storiform patterns (Fig. 2A-B). Whereas the stroma consists of more delicate spindled cells with smaller and more elongated nuclei, and less cytoplasm (Fig. 2C). The epithelial component varies between small non-keratinizing squamoid nests, as well as variably sized (tubules to cyst-like) glandular structures lined by predominantly cuboidal luminal cells and basal/myoepithelial cells with occasional squamous differentiation (Fig. 3).

**Fig. 1:**
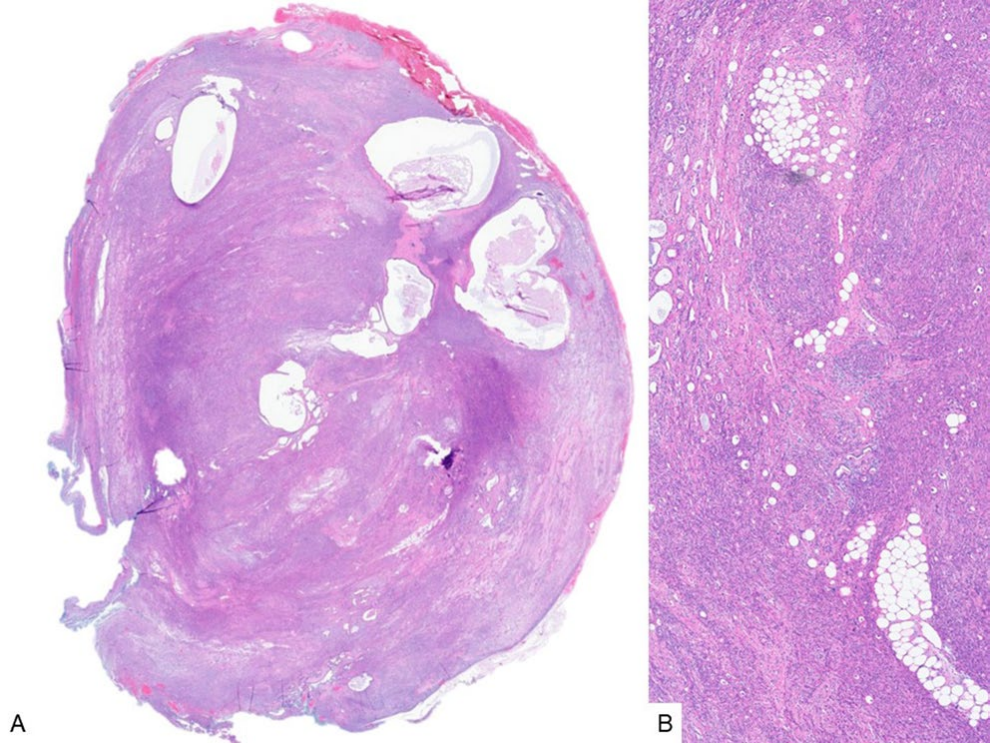
Branchioma, low-power (**A**) and medium-power (**B**) views showing a predominant stromal and spindled cell component, with scattered epithelial/tubular and mature adipose tissue elements

**Fig. 2:**
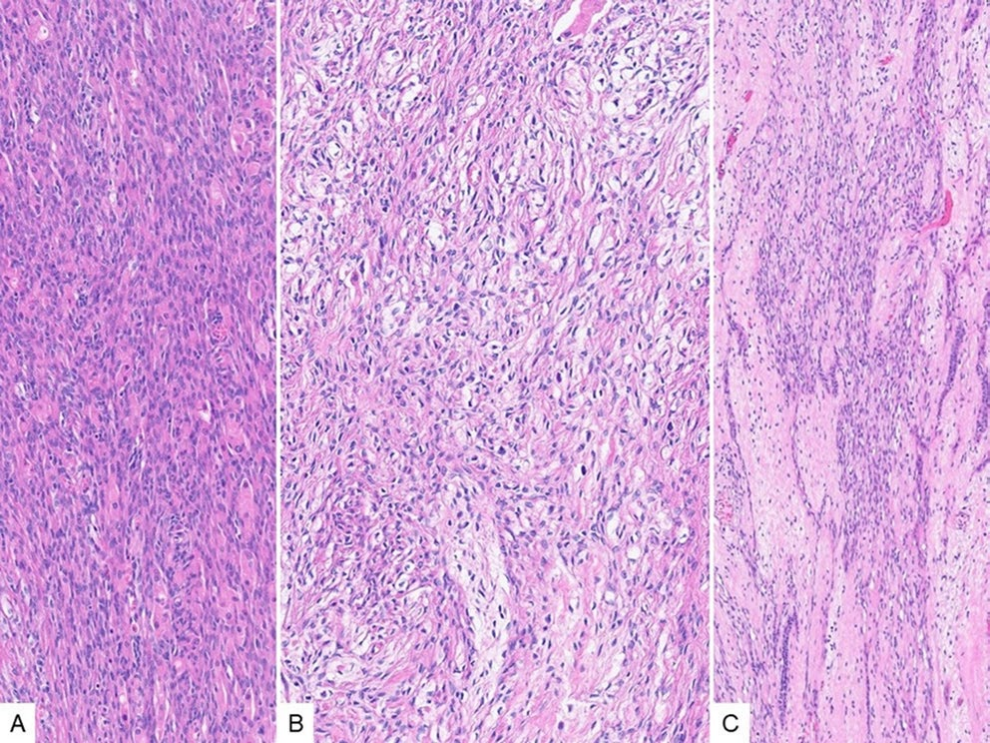
The predominant component of branchioma is comprised of plump spindled cells with large nuclei and eosinophilic (**A**) to clear (**B**) cytoplasm, arranged in fascicular or storiform patterns, present in a background of more delicate spindled stromal cells with small nuclei (C)

**Fig. 3:**
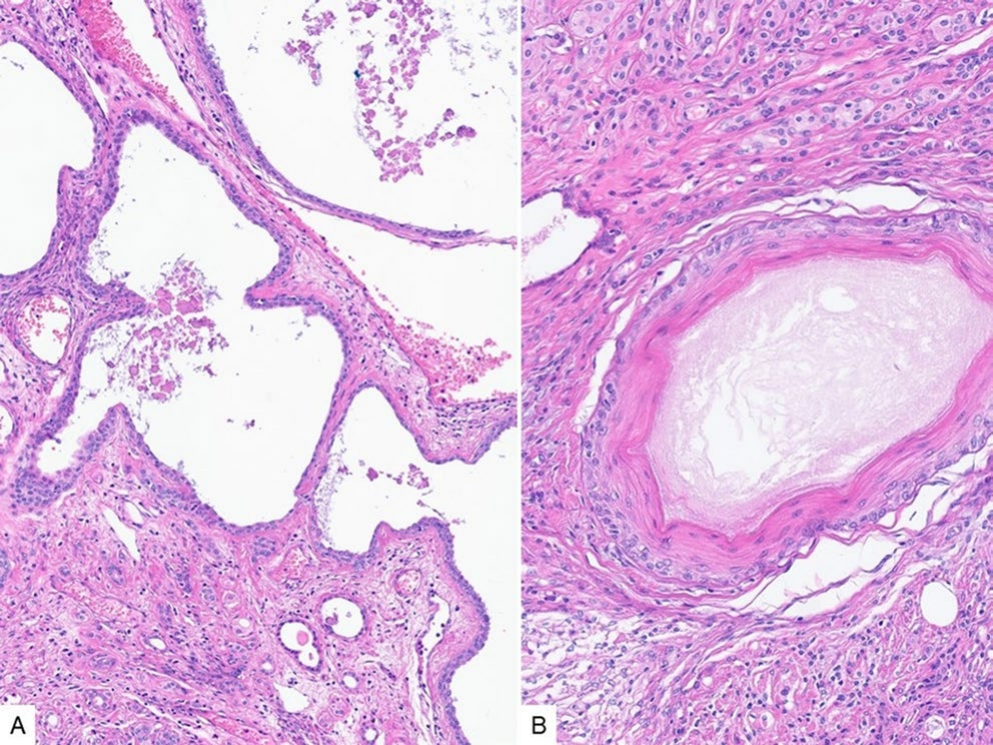
The epithelial component consists of variably sized tubules/ducts with flattened to cuboidal cell lining (**A**), which may occasionally display squamous metaplasia (**B**). Small non-keratinizing squamoid islands can also be present (B, upper third)

**Fig. 4:**
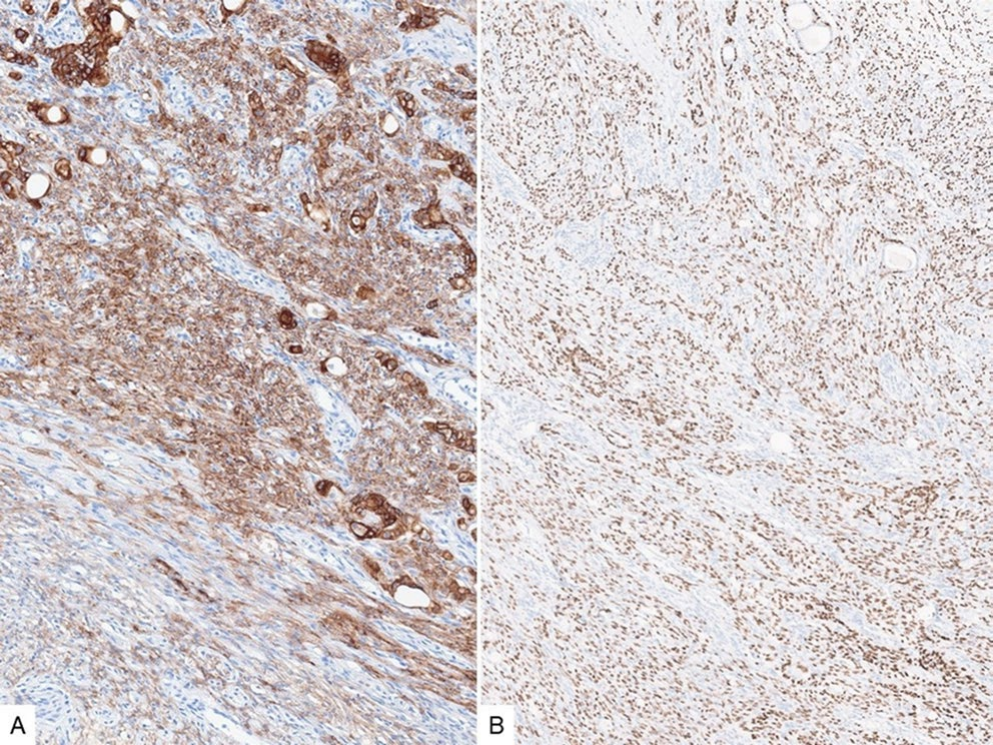
Cytokeratin AE1/AE3 (**A**) is accentuated in the epithelial islands and stains variably the spindled component. p63 (**B**) is positive in the plump/larger spindled cells as well as the basal cells of the epithelial/ductal component

**Fig. 5:**
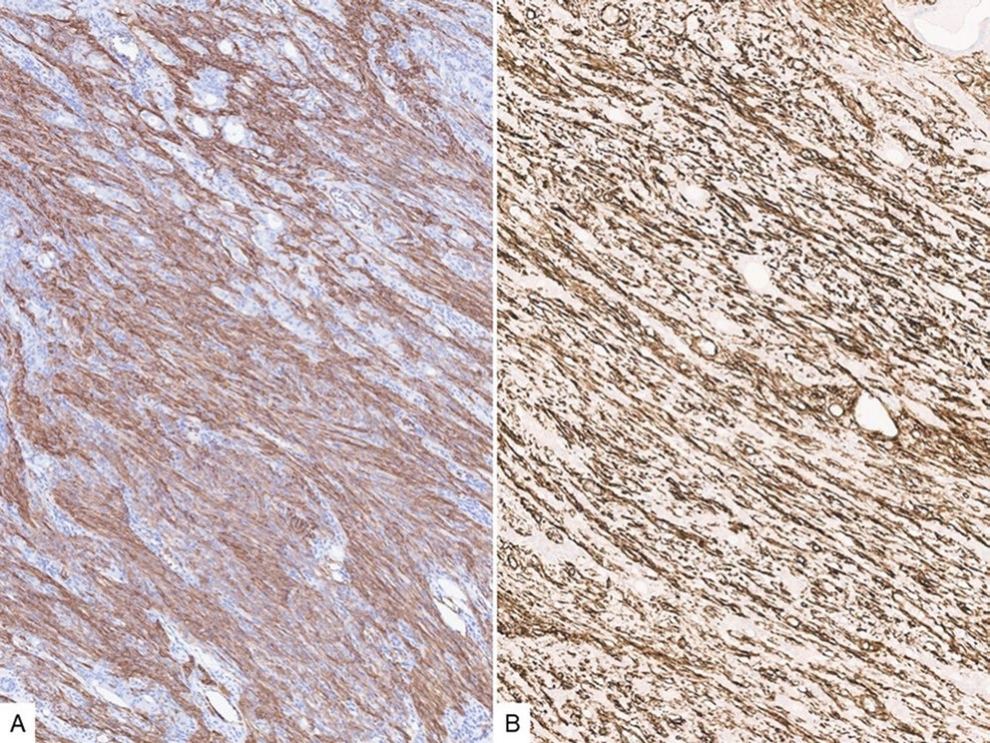
SMA (**A**) and CD34 (**B**) variably stain the spindled component, with the latter strongly highlighting the more delicate spindled cells with smaller nuclei

By immunohistochemistry, CK AE1/AE3 expression is seen in both the epithelial and spindled components but is accentuated in the former. p63 highlights the squamoid nests, the basal cells in the ductal component along with the plump spindled cells (Fig. 4). SMA and CD34 are variably positive in the spindled component, with CD34 more strongly expressed in the delicate stromal cells (Fig. 5). The immunoreactivity to cytokeratin, p63 and SMA supports at least partial myoepithelial differentiation of the spindled cells. Of note, loss of RB1 expression by immunohistochemistry was identified recently in the majority of branchiomas, similar to spindle cell lipoma, which is also commonly found in the neck.

While branchiomas are benign neoplasms, they rarely give rise to carcinomas [3]. In this case, no malignant component was identified.

Given the mixed epithelial and myoepithelial elements raises the possibility of a mixed tumor (previously known as chondroid syringoma), however, branchiomas consistently lack *PLAG1* gene alteration, and their distinct histomorphology, immunophenotype, and predilection to the suprasternoclavicular area are in keeping with a distinct entity containing biphasic primordial layers; mesodermal and endodermal.

## Data Availability

No datasets were generated or analysed during the current study.
